# The tea plant reference genome and improved gene annotation using long-read and paired-end sequencing data

**DOI:** 10.1038/s41597-019-0127-1

**Published:** 2019-07-15

**Authors:** Enhua Xia, Fangdong Li, Wei Tong, Hua Yang, Songbo Wang, Jian Zhao, Chun Liu, Liping Gao, Yuling Tai, Guangbiao She, Jun Sun, Haisheng Cao, Qiang Gao, Yeyun Li, Weiwei Deng, Xiaolan Jiang, Wenzhao Wang, Qi Chen, Shihua Zhang, Haijing Li, Junlan Wu, Ping Wang, Penghui Li, Chengying Shi, Fengya Zheng, Jianbo Jian, Bei Huang, Dai Shan, Mingming Shi, Congbing Fang, Yi Yue, Qiong Wu, Ruoheng Ge, Huijuan Zhao, Daxiang Li, Shu Wei, Bin Han, Changjun Jiang, Ye Yin, Tao Xia, Zhengzhu Zhang, Shancen Zhao, Jeffrey L. Bennetzen, Chaoling Wei, Xiaochun Wan

**Affiliations:** 1 State Key Laboratory of Tea Plant Biology and Utilization, Anhui Agricultural University, Hefei, 230036 China; 2 BGI–Shenzhen, Shenzhen, 518083 China; 3 National Center for Gene Research, Shanghai Institute of Plant Physiology and Ecology, Shanghai Institutes for Biological Sciences, Chinese Academy of Sciences, Shanghai, 20032 China; 4 Department of Genetics, University of Georgia, Athens, GA30602 USA

**Keywords:** Genome, DNA sequencing, Genome assembly algorithms, Plant genetics

## Abstract

Tea is a globally consumed non-alcohol beverage with great economic importance. However, lack of the reference genome has largely hampered the utilization of precious tea plant genetic resources towards breeding. To address this issue, we previously generated a high-quality reference genome of tea plant using Illumina and PacBio sequencing technology, which produced a total of 2,124 Gb short and 125 Gb long read data, respectively. A hybrid strategy was employed to assemble the tea genome that has been publicly released. We here described the data framework used to generate, annotate and validate the genome assembly. Besides, we re-predicted the protein-coding genes and annotated their putative functions using more comprehensive omics datasets with improved training models. We reassessed the assembly and annotation quality using the latest version of BUSCO. These data can be utilized to develop new methodologies/tools for better assembly of complex genomes, aid in finding of novel genes, variations and evolutionary clues associated with tea quality, thus help to breed new varieties with high yield and better quality in the future.

## Abstract (summary)

**Table:** 

Design Type(s)	sequence assembly objective • sequence annotation objective
Measurement Type(s)	whole genome sequencing assay • BAC • transcription profiling assay
Technology Type(s)	DNA sequencing • RNA sequencing
Factor Type(s)	developmental stage
Sample Characteristic(s)	Camellia sinensis var. sinensis • leaf • apical bud • stem • flower • fruit • root

Machine-accessible metadata file describing the reported data (ISA-Tab format)

## Background & Summary

Tea is the oldest and most prevalent nonalcoholic beverage in the world^1,2^. It is made from the cured leaves of tea plant (*Camellia sinensis*), an important economic crop planted worldwide. Tea harbors rich characteristic compounds (e.g. tea polyphenol, theanine and caffeine) that are beneficial to the human body and can effectively prevent the occurrence of malignant tumors and reduce the occurrence of cardiovascular and cerebrovascular diseases and diseases of the nervous system^3^. At present, nearly 3 billion people in more than 160 countries drink tea^2^. This makes tea an important source of economic income for the world’s major tea producing countries. With more than 18 billion cups of tea consumed daily^4^, tea is now commercially cultivated on more than 4.10 million hectares of land on a continent-wide scale, and 5.95 million metric tons of tea worldwide were produced annually in 2016.

The tea plant primarily includes two varieties namely: *C*. *sinensis* var. *sinensis* (Chinese type tea; CSS) and *C*. *sinensis* var. *assamica* (Assam type tea; CSA). The Chinese type tea accounts for over 80% of tea production worldwide and is suitable for the manufacture of six major teas. It has a broader and distinct geographical distribution from the Assam tea that is predominantly distributed in the southwest of China and the Assam region of northeast India^5^. Over the past 10 years, we have made a series of attempts to gain a fundamental understanding of the genetic basis of tea quality—an essential question that has puzzled the tea scientific community and seriously hindered the sustainable development of the tea industry for more than 50 years. We therefore initiated a joint collaborative project to generate a reference-quality genome assembly for the Chinese type tea plant^6^. In comparison with the previously published CSA genome^7^, we found that the two varieties of tea plants (CSS and CSA) diverged from their common ancestor ~0.38–1.54 million years ago. Tea plant undergoes two whole genome duplication events that facilitate the expansion of various genes associated with the biosynthesis of secondary metabolites that determine tea quality. Most importantly, we identified and functionally validated a gene involved in theanine biosynthesis. These results and datasets provide a solid foundation for the tea community to uncover the genetic basis of tea quality and genome evolution^8^.

In this descriptor, we mainly described the plant material and full data sets generated and used to assemble, annotate and validate the tea plant reference genome: (1) raw Illumina whole genome sequencing (WGS) data for genome assembly; (2) raw PacBio sequencing data for genome assembly; (3) raw PacBio RNA sequencing data from mixed tissues of tea plant for gene annotation; (4) eighteen bacterial artificial chromosomes (BACs) and BAC end sequences used for quality validation of genome assembly; and (5) the final assembly and latest release of reference genome of tea plant. We reassessed the quality of genome assembly by using datasets from the latest version of BUSCO. Additionally, we re-annotated the protein-coding genes and predicted their putative functions using more comprehensive omics datasets and improved *ab intio* prediction models. The described sequencing data and newly released annotations in this study will not only help computational biologist to test the novel methodologies and tools to assemble the complex tree genomes, but also facilitate the tea community to better understand the genetic basis of tea quality and genome evolution.

## Methods

### Selection of the target tea plant individual for genome sequencing

**Fig. 1:**
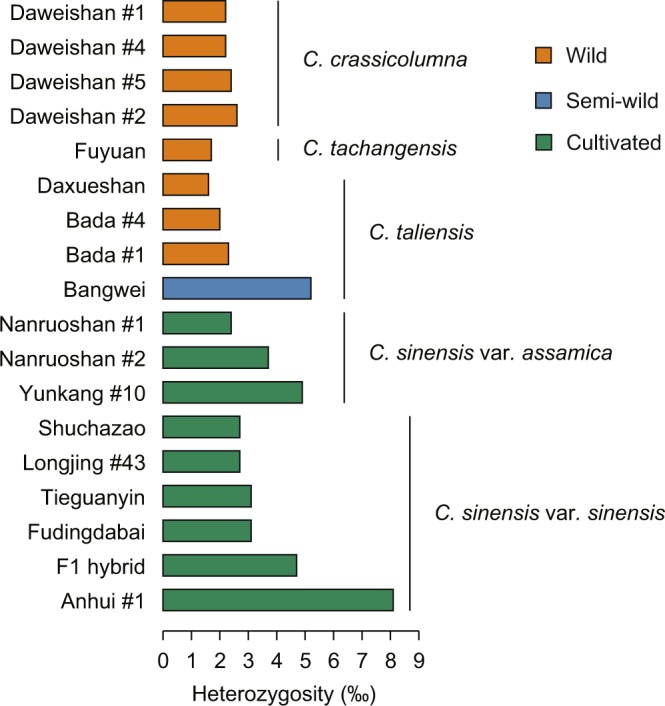
Evaluation of the heterozygosity of 18 representative tea plants using RAD-seq for selection of individuals to genome sequencing. The left panel indicates the accession names of tea plant species/varieties. The F1 individual was a hybrid from “Yunkang #10 × Fudingdabaicha”. The middle panel shows the heterozygosity dynamics among different tea plants. Orange bar represents wild tea plants, while blue and green bars characterize semi-wild and cultivated tea plants, respectively. The right panel indicates species name. The heterozygous data of each tea plant was collected from our previous work^6,10^.

Interspecific hybridization is an extensive phenomenon in tea plant, which causes large difficulties in the genome assembly^9^. In order to select the most appropriate and suitable tea plant material for sequencing, we collected a total of 18 tea plants from different locations of China that represents the majority of tea cultivars and wild relatives^10^. Fresh leaves of each individual plant were used for DNA extraction. All individuals were genotyped to estimate the degree of heterozygosity using the high-throughput genome-wide restriction site-associated DNA sequencing (RAD-Seq) technology. Only the bi-allelic SNPs were retained and used for subsequent analysis. The heterozygosity rate was estimated by the ratio of the numbers of heterozygous SNPs to the total length of the shared SNP-associated genome fragments^10^. Results showed that the heterozygosity rate of the investigated tea plants ranged from 0.0016 to 0.0081 with an average of 0.0032 (Fig. 1). We found that the commercial variety “Shuchazao” (Accession: GS2002008) exhibits a comparatively low level of heterozygosity (2.7‰) among the Chinese type tea cultivars tested, and therefore was selected for genome sequencing. Shuchazao is among the most widely grown tea plants in China with various excellent agricultural traits (e.g. early sprouting, highly resistance to cold and drought, and high yields).

### Plant material, DNA extraction, library construction and Illumina sequencing

Plant material was grown under field conditions at 916 tea plantation of Shucheng, Anhui province, China. Tender shoots were harvested and stored at −80 °C prior to DNA extraction. Cetyltrimethyl ammonium bromide (CTAB) method was employed to isolate the genomic DNA^11^. A total of twenty paired-end libraries, including ten short-insert (170 bp, 250 bp, 500 bp and 800 bp) and ten long-insert libraries (2 kb, 5 kb, 10 kb, 20 kb and 40 kb), were prepared separately following the Illumina’s protocol^6^. At least 5 μg of genomic DNA was used for the construction of small-insert paired-end and approximately 10–30 μg was used to prepare large-insert mate-pair library. The libraries were sequenced using Illumina Hiseq 2500 platform. In total, approximate 2,124 Gb of raw sequencing data, accounting ~699-fold coverage of whole genome, was generated (Table 1). The data from short-insert (<1 kb) and long-insert libraries (≥2 kb) were 1,262 Gb and 862 Gb, respectively.

**Table 1:** Summary of genome sequencing data of tea plant using Illumina and PacBio SMRT sequencing platforms.

Library Type	Insert Size (bp)	Sequencing Platform	Read Length (bp)	Number Libraries/Cells	Raw Data		Clean Data	
					Total Data (Gb)	Sequence Coverage (×)	Total Data (Gb)	Sequence Coverage (×)
**Illumina short reads**								
Paired-End	170	Hiseq 2500	150	2	209.12	68.79	192.18	63.22
	250	Hiseq 2500	150	2	456.74	150.24	361.31	118.85
	500	Hiseq 2500	90	3	356.08	117.13	305.03	100.34
	800	Hiseq 2500	90	3	239.81	78.88	189.52	62.34
Mate-Pair	2000	Hiseq 2500	90	2	119.71	39.38	62.22	20.47
	5000	Hiseq 2500	50	1	68.29	22.46	18.73	6.16
	10000	Hiseq 2500	90	3	224.10	73.72	87.26	28.70
	20000	Hiseq 2500	90	2	177.70	58.45	66.01	21.71
	40000	Hiseq 2500	90	2	272.21	89.54	42.57	14.00
Total				20	2123.76	698.59	1324.83	435.79
**PacBio SMRT long reads**								
RSII-10 kb	10000	RS II sequencer	6440	44	33.20	10.92	22.87	7.52
RSII-20 kb	20000	RS II sequencer	12632	97	92.20	30.33	63.53	20.90
Total				141	125.40	41.25	86.40	28.42

The architecture of sequencing data was summarized from our previous reported tea plant genome^6^. The estimated genome size of 3.08 Gb was used to calculate the sequence coverage of each library^6^.

### Illumina reads preprocessing and genome properties

Similar to the procedures described previously^6^, the raw sequencing data was preprocessed to remove adapter contaminations, PCR duplicates and sequencing errors. Briefly, (1) we removed the reads from short and long insert-size libraries if they separately contain more than 2% and 5% of unidentified bases (Ns); (2) we removed the reads from short and long insert-size libraries if they host more than 40% and 30% of low quality bases (phred score ≤ 7), respectively; (3) we trimmed the adapter in the reads by aligning them to adapter sequence (allowing ≤ 3 bp mismatch); (4) we removed the reads derived from PCR duplication if two paired-end reads were completely identical; (5) we aligned the sequencing reads against NCBI NR database and removed the potential contaminations derived from known bacteria or viruses. After filtering, we obtained a total of 1,325 Gb of high-quality reads, covering approximately ~436-fold of tea plant genome (Table 1).

**Fig. 2:**
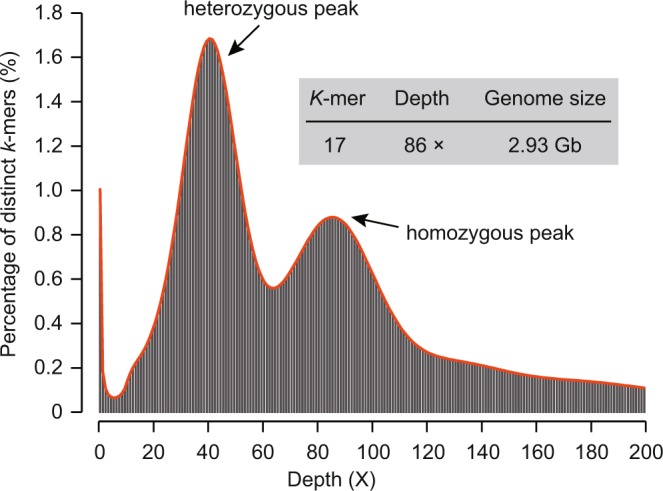
The 17-mer distribution used for the estimation of genome size of tea plant. The distribution of 17-mer was calculated using jellyfish based on the sequencing data from short insert size libraries (insert size = 500 bp). The heterozygous and homozygous peaks of read depth were marked, suggesting a high complexity of tea plant genome.

The genome properties of tea plant were characterized by performing the *k*-mer analysis of sequencing data from short-insert libraries using Jellyfish^12^. A *k*-mer refers to an oligonucleotide of *k* bp in length. The bimodal distribution of the 17-mer indicates that tea plant harbors a heterozygous diploid genome (Fig. 2) with the homozygous peak located at 86× and the heterozygous peak was found at 43×. Notably, compared to the homozygous peak on the right, the sharp heterozygous peak on the left indicates a high level of genome heterozygosity. As proposed by Liu and colleagues^13^, the genome size can be basically inferred from the total number of *k*-mers divided by the *k*-mer depth. This estimated the genome size of tea plant to be 2.93 Gb (Fig. 2), which is quite consistent with the estimation from flow cytometry (2.98 Gb)^6,7^.

### PacBio single-molecule real-time (SMRT) sequencing

Two types of PacBio libraries, including 10 kb and 20 kb library, were constructed for sequencing using PacBio RSII platform. For 10 kb libraries, the raw sequencing data was generated from 44 SMRT cells and further filtered using the RS_Subreads protocol (i.e., minimum subread length = 2 kb, minimum polymerase read quality = 0.8), resulting in a total of 33.2 Gb (~11-fold coverage) useable data (total number of subreads = 6.2 M reads, mean subread length = 5.3 kb, subread N50 = 6.4 kb). For 20 kb library, data from 97 SMRT cells were treated as described above, yielding in a total of 92.2 Gb (~30-fold coverage) data (total number of subreads = 10.6 M reads, mean subread length = 8.7 kb, subread N50 = 12.6 kb). The obtained reads were then further corrected by using the Pacbio corrected reads pipeline (PBcR). This exercise finally yielded a total of 86.4 Gb high quality subreads for subsequent data analysis (Table 1).

### Genome assembly using illumina and PacBio sequencing data

We employed a hybrid approach that integrates Illumina paired-end and PacBio long-read sequencing data to assemble the tea plant genome^6^. We *de novo* assembled the high-quality Illumina sequencing reads into contigs and scaffolds using SOAP*denovo* and Platanus^14^. Platanus is an effective *de novo* assembler for highly heterozygous genomes. Briefly, we removed the sequencing reads with 17-mer frequency ≤ 10 and merged paired-end reads from two libraries (170 bp & 250 bp) by sequence overlap, independently. This resulted in a total of 553 Gb of clean short read data used for contig construction. The contigs were constructed using SOAP*denovo*. Low coverage links and bubble structures caused by heterozygosity or errors were removed. All clean reads from small-insert and large-insert size libraries were aligned onto the preassembled contigs. According to the order and distance information, the assembled contigs were further elongated and eventually combined into scaffolds using Platanus^14^.

We closed the gaps that might be repeat sequences masked during the construction of scaffolds using Krskgf and Gapclose^15^. Briefly, all paired-end sequencing reads were first mapped onto the assembled scaffolds, and then those read pairs with one read well-aligned on the contigs and another located in the gap region were retrieved and assembled locally to close the gaps. To fill the gap as much as possible, we further applied Pbjelly^16^ to close remaining gaps within scaffolds using PacBio long-read sequencing data from 10 kb and 20 kb libraries.

Haplomerger^17^ was used for merging and truncating of scaffolds originating from heterozygous genomic regions. The resulting final assembly of tea plant genome contains 2.89 Gb contigs (N50 = 67.07 kb) and 3.14 Gb scaffolds (N50 = 1.39 Mb) (Table 2). The largest scaffold was observed 7.31 Mb in length.

**Table 2:** Statistics of the tea plant genome assembly and improved annotation.

**Assembly**	
Estimated genome size (Gb)	3.08
Number of scaffolds	14,051
Total length of scaffolds (bp)	3,141,536,798
N50 of scaffolds (bp)	1,397,810
N90 of scaffolds (bp)	358,724
Longest scaffold (bp)	7,310,916
Number of contigs	94,321
Total length of contigs (bp)	2,893,782,109
N50 of contigs (bp)	67,068
N90 of contigs (bp)	14,057
Longest contig (bp)	538,748
Gap sequence (bp)	247,754,689
Predicted coverage of the assembled sequences (%)	95.07
GC content of the genome (%)	37.84
**Annotation**	
Number of predicted protein-coding genes	53,512
Average gene length (bp)	3,747
Mean exon length (bp)	284
Average exon per gene	4.5
Mean intron length (bp)	712
Annotated to Swissport	34,694 (64.83%)
Annotated to PFAM	39,889 (74.54%)
Annotated to TAIR (version 10)	38,952 (72.79%)
Annotated to GO	21,961 (41.04%)
Annotated to KOG	14,587 (27.26%)
tRNAs	597
rRNAs	2,838
snRNAs	416
miRNAs	355
Masked repeat sequence length (bp)	1,861,774,995
Percentage of repeat sequences (%)	64.42

The statistics of genome assembly are based on sequence lengths that are larger than 1 kb. The protein-coding genes were re-predicted based on the improved *ab intio* training models and manual filtering. Putative functions of the re-annotated tea plant genes were predicted by aligning them against Swiss-Prot, InterPro, KEGG and GO databases. The statistics of genome assembly, noncoding RNAs and repeat contents were summarized from our previous work^6^.

### Genome annotation

We used a combined method that integrates *ab inito* gene prediction, homolog searching and EST/unigene-based prediction to re-annotate the protein-coding genes in the tea plant genome^6^. (1) We applied Augustus and SNAP to perform *de novo* gene prediction^18^. Compared to the previous annotation, there are two improvements in this step. We improved the accuracy and sensitivity of the prediction model applied by Augustus through iteratively self-training with full-length transcripts of tea plant. These full-length transcripts were generated by Pacbio sequencing of eight developmental tissues: apical buds, young leaves, mature leaves, old leaves, immature stems, flowers, young fruits, and tender roots^19^. Moreover, we incorporated gene predictions from SNAP, a fast and widely used gene predictor for both eukaryotic and prokaryotic gene annotation. *Arabidopsis thaliana* was set as the training organism for SNAP gene model prediction. In total, 52,987 and 126,067 gene models are annotated by Augustus and SNAP gene predictors, respectively. (2) We performed homology-based gene predictions by using the homologous sequences from proteomes of kiwifruit (*Actinidia chinensis*), coffee (*Coffea canephora*), poplar (*Populus trichocarpa*) and grape (*Vitis vinifera*), as previously described^6^. Briefly, the homologous protein sequences were first mapped to the masked assembly of tea plant genome using TBLASTN. BLAST hits were filtered by E-value (threshold 1e^−5^)^20^. The homologous genomic fragments of the target genes together with their 3 kb flanking sequences were then extracted and fed to GeneWise^21^ to define gene models using the matching proteins. This predicted a total of 59,739, 42,217, 65,800, and 40,491 tea plant gene models from kiwifruit, coffee, poplar, and grape protein alignments, respectively. (3) We performed the EST-based gene prediction by aligning a total of 26,046 tea ESTs from GenBank to genome assembly using BLAT^22^. The alignments with identity ≥90% and coverage ≥90% were retained and further fed to PASA (Program to Assemble Spliced Alignments) software^23^ to predict a total of 26,318 gene models. Besides, the aforementioned RNA-seq data from eight tissues was assembled using the StringTie^24^, generating a total of 61,681 gene models. (4) The gene models generated from different prediction methods were combined into a consensus gene set using MAKER^25^ with further filtering, yielding a total of 53,512 protein-coding genes in tea plant genome (Table 2). The average gene length is 3,747 bp with an average intron length of 712 bp.

**Fig. 3:**
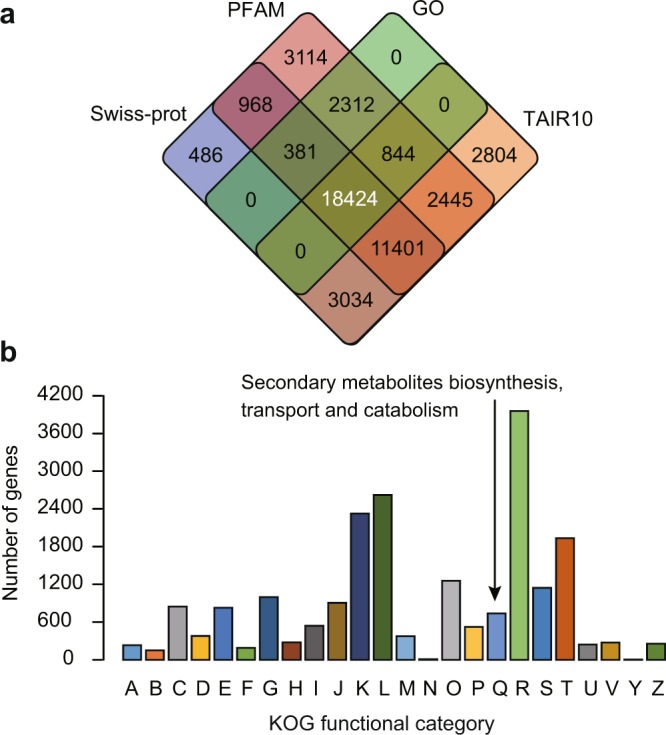
Functional annotation of the tea plant protein-coding genes. (**a**) Venn diagram shows the shared and unique annotations among Swiss-prot, PFAM, GO and The *Arabidopsis* Information Resource (TAIR; version10). (**b**) Functional classification of tea plant genes using KOG database. The functional categories of KOG are abbreviated. A: RNA processing and modification; B: chromatin structure and dynamics; C: energy production and conversion; D: cell cycle control, cell division, chromosome partitioning; E: amino acid transport and metabolism; F: nucleotide transport and metabolism; G: carbohydrate transport and metabolism; H: coenzyme transport and metabolism; I: lipid transport and metabolism; J: translation, ribosomal structure and biogenesis; K: transcription; L: replication, recombination and repair; M: cell wall/membrane/envelope biogenesis; N: cell motility; O: posttranslational modification, protein turnover, chaperones; P: inorganic ion transport and metabolism; Q: secondary metabolites biosynthesis, transport and catabolism; R: general function prediction only; S: function unknown; T: signal transduction mechanisms; U: intracellular trafficking, secretion, and vesicular transport; V: defense mechanisms; Y: nuclear structure; and Z: cytoskeleton.

We predicted the putative functions of the 53,512 tea plant genes using various public well-known protein databases (Fig. 3a). We searched homologous of the tea plant genes in the Swiss-prot protein databases using BLASTP^20^ with an E-value threshold of ≤10^−5^. In total, the function of 34,694 (64.83%) genes are annotated (Table 2). We used InterProScan^26^ to detect conserved domains and assign gene ontology (GO) terms to tea plant genes. Results show that a total of 39,889 and 21,961 genes can be separately assigned with domains and GO terms. We aligned the tea plant genes against *A*. *thaliana* protein datasets (version 10), and found that 38,952 tea plant genes can be allocated functions. Furthermore, we functionally classified the tea plant genes using EuKaryotic Orthologous Groups (KOG). Results demonstrated that a total of 14,587 (27.26%) genes could be annotated with KOG functions. In particular, 831 genes were predicted to be involved in secondary metabolite biosynthesis, transport and catabolism (Fig. 3b).

The transposable elements (TEs) were annotated using a combination of *de novo* methods and homology-based methods as described previously^6^. We performed *de novo* prediction using RepeatModeler and LTR-Finder^27^. The repetitive sequences yielded from these two programs were combined to a non-redundant repeat sequence library. With this library, we scanned the representative sequences in tea plant genome. We also performed the homology-based prediction by using RepeatMasker and RepeatProteinMask based on the repeat library from Repbase database^28^ (Repbase-18.04). TEs annotated from *de novo* and homology-based methods were combined into a single dataset. The redundancies between them were removed to generate the final TEs. The identified TEs were then clustered into families according to their sequence identity. We also employed Tandem Repeats Finder (TRF)^29^ to identify tandem repeats in the tea plant genome. In total, we identified 1.86 Gb (64%) of repetitive sequences in tea plant genome (Table 2), which is comparable to the proportion of TEs identified in Sanger-derived BACs (56.81%)^30^. The above methods are expanded versions of descriptions in our related work^6,10^.

## Data Records

Raw Illumina sequencing reads are available at the National Center for Biotechnology Information (NCBI) Sequence Read Archive (SRA) database under Accession Number SRP099527^31^. The raw SMRT PacBio whole genome sequencing data can be downloaded from NCBI SRA database under Accession numbers SRR8334869 and SRR8334870^32^. The raw data of tea plant BAC end sequences are deposited in the NCBI SRA database and can be freely accessed under the Accession Numbers SRP111069^33^. The raw data of SMRT sequencing of eight tea plant tissues can be downloaded from NCBI SRA database under Accession Numbers SRX2748122^34^. The final genome assembly and gene annotation are deposited into NCBI GenBank database under the Accession Number SDRB00000000^35^. The improved gene prediction (GFF3), coding and peptide sequences (FASTA), and gene functional annotations (TXT) are uploaded into Figshare^36^, and can also be accessed from the newly developed Tea Plant Information Archive database (TPIA; http://tpia.teaplant.org)^8^.

## Technical Validation

Compared to the previous evaluations^29^, we reassessed the completeness of genome assembly and quality of re-annotated gene models using the latest version of Benchmarking Universal Single-Copy Orthologs (BUSCO)^37^. We ran BUSCO using a total of 1,440 orthologous groups from plant lineages. *Arabidopsis* was employed as model for training during Augustus gene model prediction^18^. The results showed that the majority of the plant core genes/orthologues (95.5%) was present in the tea plant genome assembly (Table 3). Among them, approximately 92.4% and 3.1% were identified as complete and fragmented, respectively. This indicates a relative complete genome assembly.

**Table 3:** Validation of the assembly quality and improved gene annotation of tea plant genome using three methodologies.

Validation of assembly quality	Number	Percentage (%)
**BUSCO validation**		
Total BUSCO groups	1,440	100
Complete single-copy BUSCOs	1,180	81.9
Complete duplicated BUSCOs	151	10.5
Fragmented BUSCOs	44	3.1
Missing BUSCOs	65	4.5
**BAC validation**		
Total BACs (#)	18	100
Total length (bp)	2,080,846	100
Aligned BACs (bp)	1,182,063	98.30
**PCR validation**		
Total PCR experiments	24	100
Success PCR experiments	22	91.67
**Validation of gene prediction**		
Total BUSCO groups	1,440	100
Complete single-copy BUSCOs	1,068	74.2
Complete duplicated BUSCOs	173	12.0
Fragmented BUSCOs	118	8.2
Missing BUSCOs	81	5.6

The completeness of genome assembly and gene re-annotation were evaluated using the latest version of BUSCO (v3.0.2). The result of BAC alignment and PCR validation were summarized from our previous reported tea plant genome^6^.

We also used the 1,440 BUSCO conserved genes from plant lineage to assess the quality of gene models re-predicted in this study. Results showed that nearly 94.4% of the plant conserved genes/orthologues can be found in the predicted gene set, evidencing a good quality of the gene annotation. The complete and fragmented BUSCO account for 86.2% and 8.2% of the total plant core gene/orthologue groups (Table 3).

As described previously^6^, we fully sequenced a total of 18 BACs to assess the accuracy of genome assembly. The BACs were randomly selected from a constructed BAC library that consists of 161,280 clones covering approximately 6-fold depth of tea plant genome^30^. Results show that 98.3% of the sequenced BACs can be well aligned to the genome assembly, showing a high base-level accuracy of the genome assembly (Table 3). We further employed a total of 24 PCR reactions to investigate the quality of genome assembly. The primers were provided in the previously published tea plant genome paper^6^. Results showed that, of the 24 primer pairs, 22 (91.67%) could yield approximately same size of PCR fragment, showing an accurate genome assembly at the base resolution (Table 3).

These validations are expanded versions of descriptions in our related work^6,30^.

## ISA-Tab metadata file

Download metadata file

## Data Availability

All the bioinformatics tools/packages used in building of tea plant genome are described below along with their versions, settings and parameters. (**1**) **Jellyfish**: version 2.2.6, k-mer size of 23; (**2**) **RS_Subreads protocol**: minimum subread length (equal or larger than 2 kb); minimum polymerase read quality (equal or larger than 0.8); (**3**) **Platanus**: version 1.24, default parameters, minimum number of link was 4 for scaffolding; (**4**) **Krskgf**: version 1.2, default parameters; (**5**) **GapCloser**: version 1.12, default parameters; (**6**) **Pbjelly**: version 15.8.24, default parameters; (**7**) **Haplomerger**: version 20161205, default parameters, “–step = 20” to speed up the LASTZ process; “minOverlap = 99,999,999” to avoid mixing up haplotypes; (**8**) **Augustus**: version 3.0.3, parameters: –gff3 = on, –species = teaplant, –genemodel = partial, –UTR = off, –uniqueGeneId = true; (**9**) **Blastall**: version 2.2.26, parameters: -e 1e-5, -F F; (**10**) **GeneWise**: version 2.2.0, parameters: -genesf -quiet –pseudo; (**11**) **PASA**: version 2.0.2, parameters: -C; “-R” to run alignment/assembly pipeline; “-T” to indicate that transcripts were trimmed using the TGI seqclean tool; (**12**) **StringTie**: version 1.3.1b, default parameters; (**13**) **MAKER**: version 2.31.8, parameters: organism_type = eukaryotic, min_contig = 150, min_protein = 50, alt_splice = 1, correct_est_fusion = 1; (**14**) **InterProScan**: version 5.3–46.0, parameters:–disable-precalc,–goterms,–pathways; (**15**) **RepeatModeler**: version 1.0.11, parameters: -database teaplant, -e ncbi, -pa 30; **16**) **LTR_Finder**: version 1.07, default parameters; (**17**) **Repeatmasker**: version 4.0.5, parameters: -e ncbi, -pa 30; (**18**) **RepeatProteinMask**: version 4.0.5, default parameters; (**19**) **Tandem Repeats Finder**: version 4.04, default parameters; (**20**) **BUSCO**: version 2.0, default parameters, the lineage dataset is: embryophyta_odb9 (Creation date: 2016-11-01, number of species: 30, number of BUSCOs: 1440); (**21**) **Primer premier**: version 5.0.
